# The Preventive Effects and the Mechanisms of Action of Navel Orange Peel Hydroethanolic Extract, Naringin, and Naringenin in N-Acetyl-p-aminophenol-Induced Liver Injury in Wistar Rats

**DOI:** 10.1155/2019/2745352

**Published:** 2019-03-26

**Authors:** Osama M. Ahmed, Hanaa I. Fahim, Heba Y. Ahmed, Hessah Mohammed Al-Muzafar, Rasha R. Ahmed, Kamal Adel Amin, El-Shaymaa El-Nahass, Walaa H. Abdelazeem

**Affiliations:** ^1^Physiology Division, Zoology Department, Faculty of Science, Beni-Suef University, Beni-Suef, Egypt; ^2^Rodents Division, Department of Harmful Animals, Plant Protection Research Institute, Agriculture Research Center, Egypt; ^3^Chemistry Department, College of Science, Imam Abdulrahman Bin Faisal University, P.O. Box 1982, Dammam 31441, Saudi Arabia; ^4^Cell Biology, Histology and Genetics Division, Zoology Department, Faculty of Science, Beni-Suef University, Beni-Suef, Egypt; ^5^Department of Pathology, Faculty of Veterinary Medicine, Beni-Suef University, Beni-Suef, Egypt

## Abstract

N-Acetyl-p-aminophenol (APAP) or acetaminophen is the most common drug ingredient worldwide. It is found in more than 600 different over-the-counter and prescription medicines. Its long-term and overdose use is highly toxic and may result in liver injury. Thus, this study was designed to assess the preventive effects and to suggest the mechanisms of action of the navel orange peel hydroethanolic extract, naringin, and naringenin in APAP-induced hepatotoxicity in male Wistar rats. APAP was administered to male Wistar rats at a dose level of 0.5 g/kg body weight (b.w.) by oral gavage every other day for 4 weeks. APAP-administered rats were treated with the navel orange peel hydroethanolic extract (50 mg/kg b.w.), naringin (20 mg/kg b.w.), and naringenin (20 mg/kg b.w.) by oral gavage every other day during the same period of APAP administration. The treatments of APAP-administered rats with the peel extract, naringin, and naringenin produced a significant decrease in the elevated serum AST, ALT, ALP, LDH, and GGT activities as well as total bilirubin and TNF-*α* levels while they induced a significant increase in the lowered serum albumin and IL-4 levels. The treatments also resulted in a significant decrease in the elevated liver lipid peroxidation and enhanced the liver GSH content and SOD, GST, and GPx activities as compared with APAP-administered control; the peel extract was the most potent in improving the liver LPO, GSH content, and GPx activity. In addition, the three treatments significantly downregulated the elevated hepatic proapoptotic mediators p53, Bax, and caspase-3 and significantly upregulated the suppressed antiapoptotic protein, Bcl-2, in APAP-administered rats. In association, the treatments markedly amended the APAP-induced liver histopathological deteriorations that include hepatocyte steatosis, cytoplasmic vacuolization, hydropic degeneration, and necrosis together with mononuclear leucocytic and fibroblastic inflammatory cells' infiltration. In conclusion, the navel orange peel hydroethanolic extract, naringin, and naringenin may exert their hepatopreventive effects in APAP-administered rats *via* enhancement of the antioxidant defense system and suppression of inflammation and apoptosis.

## 1. Introduction

The liver is the largest internal organ in the human body, and it is the chief site for intense anabolism, catabolism, and excretion [1, 2]. It plays a major role in detoxification and excretion of many exogenous and endogenous compounds; thus, any impairment of its structural integrity and functions may cause many implications on one's health [3]. The cellular necrosis, elevated lipid peroxidation, and depleted glutathione (GSH) level are associated with liver damage [4]. Moreover, the serum levels of several biomarkers such as alkaline phosphatase (ALP), transaminases, bilirubin, triglycerides, and cholesterol are increased in hepatic diseases [5]. Thus, liver damage may be a result of distortion of the metabolic functions [4, 6].

N-Acetyl-p-aminophenol (APAP), acetaminophen, or paracetamol is an extensively prescribed and over-the-counter (OTC) analgesic and antipyretic drug, and it is used either as a single agent or in combination with another drug [2, 7]. APAP is a safe drug with high therapeutic index. However, the administration of an overdose even acute or chronic can result in harmful adverse effects including liver and kidney toxicities [8]. The metabolism of the ingested therapeutic dose of APAP is mainly accomplished by cytochrome P450 followed by glucuronidation or sulfation whereas N-acetyl-p-benzoquinone imine (NAPQI) is conjugated with GSH [9]. On the other hand, a high dose saturates the detoxification pathways of APAP due to glucuronidation and sulfation insufficiency [8, 10]. Thus, excessive drug accumulations become accessible for biotransformation and metabolism by cytochrome P450, which result in GSH depletion [11]. Exhausted GSH levels allow NAPQI free to bind with other targeted cellular proteins which evoke cellular oxidative stress and cellular necrosis [12]. Reactive oxygen species (ROS) production was found to be associated with excessive and long-term APAP administration and biotransformation [13]. In this regard, many investigators stated that oxidative stress plays a pivotal role in the pathogenesis of drug-induced damage and has been implicated in the mechanisms of action that lead to necrosis [14]. Moreover, it was reported that APAP could induce organ damage by activating apoptotic death and inflammation which were manifested by an increase in the expression of caspase-3, caspase-9, protein 53 (p53), nuclear factor-kappa B (NF-*κ*B), and inducible nitric oxide synthase (iNOS) as well as by a decrease in B-cell lymphoma-2 (Bcl-2) expression [15, 16]. On the contrary, it was elucidated by other publication that p53 prevents progression of liver injury by maintaining mitochondrial and metabolic homeostasis after APAP overdose-induced acute liver injury [17].

Many plant extracts have been shown to possess hepatoprotective activities [18–20]. *Citrus* peels are one of the main components of citrus by-products, and they are rich sources of biofunctional materials including flavonoids, *β*- and *γ*-sitosterol, coumarins, glycosides, and volatile oils [21]. Polymethoxylated flavones and flavanones, including hesperidin, naringin, and rutin, are found in both *Citrus* pulp and peel [22]. Bioflavonoids have been considered as a potent bioactive moiety against free radicals and oxidative stress [23]. Naringin and naringenin, two bioflavonoids found in *Citrus* fruit peel, have many pharmacological properties including antimicrobial, antidiabetic, and toxicity-protecting properties [24].

Several studies have demonstrated that oxidative stress, inflammation, and cell apoptosis represent key elements in the occurrence and development of organ injury including APAP-induced liver injury [25, 26]. Hence, the regulation of oxidative damage, inflammation, and apoptosis and the related cascade responses is considered to be crucial in therapeutic strategy for preventing many drug toxicities. The treatment with plant extracts and plant constituents, which have antioxidant and anti-inflammatory properties, may provide an efficient strategy to counteract the side effects of the chemical drugs.

In conduction with the previous publications, the present study is aimed at assessing the anticipated hepatopreventive effects and suggesting the probable mechanisms of action of the navel orange peel hydroethanolic extract, naringin, and naringenin in APAP-administered Wistar rats.

## 2. Materials and Methods

### 2.1. Experimental Animals

Adult male Wistar rats weighing 130-150 g (10-12 weeks) were used as experimental animals in the present work. They were obtained from the animal house in the National Research Center, Cairo, Egypt. The animals were housed in good aerated cages in the Animal House of Faculty of Science, Beni-Suef University, Egypt, at 12 hours daily under light-dark cycles and temperature between 20 and 25°C. Animals were given daily standard pelleted diet and were supplied water *ad libitum*. The animals were kept for two weeks under observation before the onset of the experiment to exclude any intercurrent infection. All animal methodologies are in agreement with the recommendations of the Experimental Animals Ethics Committee of the Faculty of Science, Beni-Suef University. The ethical Approval number is BSU/FS/2014/4.

### 2.2. Chemicals

APAP, naringin, naringenin, and carboxymethyl cellulose (CMC) were obtained from Sigma-Aldrich (St. Louis, MO, USA) through Trading Dynamic Company, Al-Haram, Giza, Egypt. All other used chemicals were of analytical grade.

### 2.3. Extract Preparation

Fruits of navel orange (a mutation of *Citrus sinensis*) were obtained from local markets in Beni-Suef Governorate. They were authenticated by Dr. Walaa A. Hasan, Assistant Professor of Plant Taxonomy and the director of the Herbarium of Botany Department, Botany Department, Faculty of Science, Beni-Suef University, Beni-Suef, Egypt. Navel orange fruits were washed several times with fresh water to ensure removal of any contamination. Then, they were peeled and the peels were air-dried in a shaded area for twenty days. The dried peels were coarsely powdered, and the powder was macerated in 70% aqueous ethanol for 3 days at room temperature. To fully mix the powder with the extraction solvent, the suspensions were allowed to be stirred frequently. The 70% ethanol extracts were then filtered through Whatman filter paper to remove ethanol and water. The filtrate was evaporated under a vacuum using a rotary evaporator to yield a hydroethanolic extract of navel orange peel [24, 27].

### 2.4. Gas Chromatography-Mass Spectrometry (GC-MS) Analysis

Chemical analysis of the navel orange peel hydroethanolic extract was performed in the Central Laboratory of the Faculty of Postgraduate Studies for Advanced Sciences, Beni-Suef University, Egypt, by using gas chromatography (GC) system 7890A/5975C inert mass spectrometry (MS) with the Triple-Axis Detector, Agilent Technologies, Germany. The constituents were identified by comparing their mass spectra with the spectra of derivatives in the Library Search Report (C:\Database\NIST11.L; C:\Database\demo.l).

### 2.5. High-Performance Liquid Chromatography- (HPLC-) Mass Spectrometry (MS) Analysis

HPLC-MS analysis of the hydroethanolic extract of navel orange peels was performed in the Central Laboratory of the Faculty of Postgraduate Studies for Advanced Sciences, Beni-Suef University, Egypt, by using the HPLC-MS system, Infinity 1260, Agilent Technologies, Germany, coupled with a diode array detector (DAD). Standards including quercetin, hesperidin, hesperetin, naringin, naringenin, diosmin, and gallic acid were used to identify the various peaks in the HPLC-MS chromatogram. The navel orange peel hydroethanolic extract was dissolved in water : methanol (80 : 20 *v*/*v*) at a concentration of 10 mg/3 ml and filtered with a 0.45 *μ*m filter, before injection of 20 *μ*l into the HPLC system. Spectral UV data from all peaks were collected in the range 240-400 nm, and chromatograms were recorded at 340 and 270 nm for phenolic compounds according to the method of Negri et al. [28].

### 2.6. Experimental Design

Thirty animals used in this study were allocated into five groups (six rats for each) designed as follows:

Group 1 (normal control group): rats of this group were regarded as normal control group and were orally administered the equivalent volume of CMC (1% *w*/*v*), every other day for 4 weeks by oral gavage.
Group 2 (APAP group): animals of this group were regarded as APAP control group and were orally administered APAP (dissolved in distilled water) at a dose level of 0.5 g/kg b.w. every other day for 4 weeks by oral gavage [29].
Group 3 (APAP + navel orange peel hydroethanolic extract group): rats of this group were administered APAP as group 2 and were treated with the navel orange peel hydroethanolic extract (dissolved in 1% CMC) at a dose 50 mg/kg b.w. [27] by oral gavage every other day for 4 weeks.
Group 4 (APAP + naringin group): rats of this group were administered APAP as group 2 and were treated with naringin (dissolved in 1% CMC) at a dose level of 20 mg/kg b.w. [30] by oral gavage every other day for 4 weeks.
Group 5 (APAP + naringenin group): rats of this group were administered APAP as group 2 and were treated with naringenin (dissolved in 1% CMC) at a dose level of 20 mg/kg b.w. [31] by oral gavage every other day for 4 weeks.

### 2.7. Blood and Liver Sampling

At the end of the experiment, blood samples were collected from the jugular vein, left to coagulate at room temperature, and then centrifuged at 3000 r.p.m. at room temperature for 15 minutes. The clear nonhaemolysed supernatant sera were quickly separated and kept frozen at -30°C until used for various biochemical investigations. After blood sampling, animals were decapitated by cervical dislocation and dissected. Livers were quickly excised. Pieces of liver (5 mm^3^) of each animal were fixed in 10% neutral buffer formalin for histopathological studies. Part of liver (0.5 g) of each rat was homogenized in 5 ml 0.9% NaCl. The homogenate was centrifuged at 3000 r.p.m. for 15 minutes, and separated supernatants were frozen at -30°C until used for detection of oxidative stress and antioxidant defense system markers.

### 2.8. Detection of Liver Function Biomarkers in Serum

Serum of alanine transaminase (ALT) and aspartate transaminase (AST) activities were assayed according to the methods of Bergmeyer et al. [32] and Gella et al. [33] by reagent kits purchased from Biosystem S.A. (Spain). Serum alkaline phosphatase (ALP) and gamma-glutamyl transferase (GGT) activities were measured according to the methods of Schumann et al. [34] and Szasz et al. [35], respectively, by reagent kits obtained from Biosystem S.A. (Spain). Lactate dehydrogenase (LDH) activity was determined according to the method of Pesce [36] by reagent kits purchased from Spinreact (Spain). Albumin and total bilirubin levels were determined according to the methods of Doumas et al. [37] and Jendrassik and Grof [38], respectively, by kits obtained from HUMAN Gesellschaft für Biochemica und Diagnostica mbH, Wiesbaden, Germany.

### 2.9. Detection of Markers of Oxidative Stress and Antioxidant Defense System in Liver

Liver lipid peroxidation (LPO) and glutathione (GSH) content were estimated according to the procedures of Beutler et al. [39] and Preuss et al. [40], respectively. Liver glutathione peroxidase (GPx), glutathione-*S*-transferase (GST), and superoxide dismutase (SOD) activities were assayed according to the principles and procedures of Matkovics et al. [41], Mannervik and Guthenberg [42], and Marklund and Marklund [43], respectively. All reagents used for detection of oxidative stress and antioxidant parameters were prepared in the laboratory from chemicals of analytical grades.

### 2.10. Tumor Necrosis Factor-*α* (TNF-*α*) and Interleukin-4 (IL-4) Assays

Serum levels of proinflammatory cytokine TNF-*α* and the anti-inflammatory cytokine IL-4 were assayed by the sandwich enzyme immunoassay based on the reported methods of Howard and Harada [44] and Croft et al. [45], respectively, by kits purchased from R&D Systems (USA).

### 2.11. Histopathological Investigations

After decapitation and dissection of rats, livers were rapidly excised and perfused in saline solution. Pieces (5 mm^3^) from liver of rats from different groups were fixed in 10% neutral buffered formalin for 24 hours. The fixed organs were washed in tap water and passaged in serial dilutions of ethyl alcohol for dehydration. Specimens were cleared in xylene and embedded in paraffin at 56°C in an oven for 24 hours. Paraffin wax tissue blocks were prepared for sectioning at 4 *μ*m thickness. The obtained tissue sections mounted on glass slides were deparaffinized and stained with hematoxylin and eosin (H&E) stains [46]. The stained liver sections were examined for detection of histological lesions. In three randomly selected fields of each section (×100), lesions or injuries were graded as absent (0), mild (I), moderate (II), and severe (III) for changes, 0%, less than 30%, 30-50%, and more than 50%, respectively [47]. The graded lesions included steatosis or fatty changes, inflammatory cell infiltration, necrosis, hydropic degeneration, vacuolar degeneration, vascular congestion, and Kupffer cell proliferation (activation).

### 2.12. Immunohistochemical Detection of p53, Bax, Caspase-3, and Bcl-2

The liver samples, fixed in 10% neutral buffered formalin, were transferred to the Department of Pathology, National Cancer Institute, for processing, blocking, and sectioning into 5 *μ*m thick sections that were mounted on positive-charged slides (Fisher Scientific, Pittsburgh, PA). The p53, Bax, caspase-3, and Bcl-2 reactivity was processed according to the methods Hussein and Ahmed [48], Galaly et al. [49], and Ahmed and Ahmed [50, 51]. In brief, after antigen retrieval, diluted primary antibodies for p53, Bax, caspase, or Bcl-2 (Santa Cruz Biotechnology, Santa Cruz, CA, USA), were incubated with liver sections for 1 hour. Diluted biotinylated secondary antibodies (DakoCytomation Kit) were added and incubation was done for 15 minutes at 37°C. Thereafter, horseradish peroxidase conjugated with streptavidin (DakoCytomation Kit) was applied for further 15-minute incubation. The bound antibody complex was visualized by the reaction of 3,3′-diaminobenzidine (DAB) substrate and counter staining with hematoxylin. All liver sections were incubated under the same conditions with the same dilutions of antibodies and at the same period, so the immunostaining was comparable among the different study groups. For each preparation, a negative control was performed (a slide without primary antibody). The slides were visualized under a light microscope and the extent of cell immunopositivity was assessed. Images of sections of the liver were captured using a digital camera (Leica, DM2500 M Leica, Wetzlar, Germany). Examination and analysis of labelling were performed using free software version ImageJ (1.51d) [52]. The ImageJ software was used to measure the integrated intensities (in pixels) of the positive reaction of p53, Bax, caspase-3, and Bcl-2.

### 2.13. Statistical Analysis

All data were expressed as means ± standard error (SE). Statistical analysis was performed using Statistical Package for Social Sciences (SPSS) computer software (version 22), IBM software, USA. A one-way analysis of variance (ANOVA) test was used to elucidate significance among group means, followed by Tukey's post hoc test and least significance difference (LSD) to compare between different groups for the same variable at *p* < 0.05.

## 3. Results

### 3.1. GC-MS Analysis of Navel Orange Peel Hydroethanolic Extract

**Figure 1:**
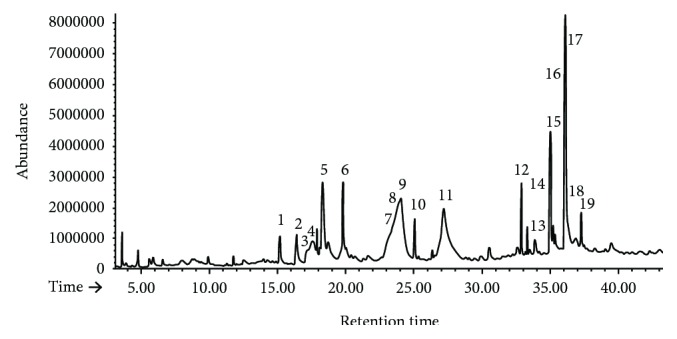
GC-MS chromatogram of the navel orange peel hydroethanolic extract.

The GC-MS analysis (Table 1 and Figure 1) indicated the presence of multiple phytochemicals. The main constituents and groups which have a concentration of more than 1% of total include delta 2-tetrazaboroline, 5-ethyl- 1,4-dimethyl-, 4,5-diamino-2-hydroxypyrimidine, thymine, 4H-pyran-4-one (a cyclic nucleus in the chemical structure of quercetin, naringenin, naringin, and hesperetin), 2,3-dihydro-3,5-di hydroxy-6-methyl-, 2-butanone, 4-hydroxy-3-methyl-, dimethylamine, N-(neopentyloxy)-, 5-hydroxymethylfurfural, 4-hexen-3-one, 4,5-dimethyl-, 2-methoxy-4-vinylphenol, 3-methoxyacetophenone, dodecane, 3-deoxy-d-mannoic lactone, beta-D-glucopyranose, 4-O-beta-D-galactopyranosyl-, sucrose, 9,12-octadecadienoic acid (Z,Z)-, *cis*-7-dodecen-1-yl acetate, oleic acid, 9-octadecenoic acid, (E)-, lupanine, stigmasterol, 1-methyl-4-(1-methylethenyl)-3-[1-methyl-1-(4-methyl-hex-5-enyl)-5,9-dimethyldec-4-enyl] cyclohexene, 26,27-dinorergosta-5,23-dien-3-ol, (3beta)-, and 9-octadecenamide, (Z)-.

5-Hydroxymethylfurfural, 4-hexen-3-one, 4,5-dimethyl-, dodecane, and lupanine have the highest percent of total.

### 3.2. HPLC-MS Analysis of Navel Orange Peel Hydroethanolic Extract

**Figure 2:**
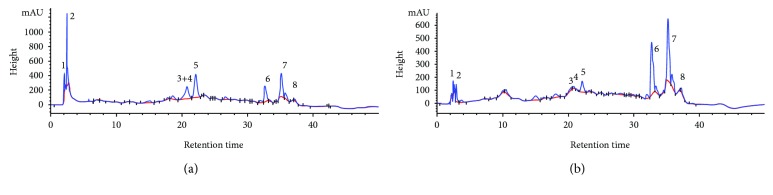
HPLC-MS fingerprint of the navel orange peel hydroethanolic extract at 270 nm (a) and at 340 nm (b) indicating the presence of diosmin (1), gallic acid (2), naringin (3), rutin (4), hesperidin (5), quercetin (6), naringenin (7), and hesperetin (8).

HPLC-MS analysis indicated in Figures 2(a) and 2(b) revealed the presence of diosmin, gallic acid, naringin, rutin, hesperidin, quercetin, naringenin, and hesperetin in the navel orange peel hydroethanolic extract. The hesperidin, quercetin, naringenin, and gallic acid derivatives are the most abundant.

### 3.3. Effects on Liver Function Parameters

Oral APAP administration caused a marked impairment in liver function as demonstrated by the significant (*p* < 0.05) increase in serum ALT, AST, ALP, LDH, and GGT activities and total bilirubin level. In contrast, APAP administration produced a significant decrease (*p* < 0.05) in serum albumin level recording a percentage of -16.09% as compared with normal. The treatment of APAP-administered rats with the navel orange peel hydroethanolic extract, naringin, and naringenin induced a significant improvement (*p* < 0.05) in serum ALT, AST, ALP, LDH, and GGT activities and albumin and total bilirubin levels as compared to APAP-administered rats (Table 2).

### 3.4. Effects on Liver Oxidative Stress and Antioxidant Defense System Parameters

As depicted in Table 3, the administration of APAP to normal rats significantly (*p* < 0.05) elevated the liver LPO (+109.56%) and significantly (*p* < 0.05) decreased level of GSH (-34.31%) and activities of GST (-23.42%), GPx (-8.49%), and SOD (-31.63%) as compared to the normal control rats. The oral administration of the navel orange peel hydroethanolic extract, naringin, and naringenin to APAP-administered rats induced a significant amelioration (*p* < 0.05) in the LPO recording percentage decreases of 35.49, 26.32, and 21.69%, respectively. Furthermore, liver GSH content was significantly (*p* < 0.05) increased as a result of the treatment with the navel orange peel hydroethanolic extract, naringin, and naringenin recording percentage increases of 31.18, 26.18, and 25.08%, respectively. These treatments also induced a significant (*p* < 0.05) increase in GPx activity. However, while liver GST activity was significantly increased (*p* < 0.05) only as a result of naringin, SOD activity was significantly increased (*p* < 0.05) as a result of the peel hydroethanolic extract and naringenin. The navel orange peel hydroethanolic extract was the most potent in increasing the depleted hepatic GSH level and GPx activity and in decreasing the elevated hepatic LPO in APAP-administered rats while naringin was the most effective in increasing the lowered GST activity in APAP-administered rats.

### 3.5. Effects on Serum TNF-*α* and IL-4 Levels

Oral administration of APAP to normal rats produced a significant increase (*p* > 0.05; 295.25%) in serum TNF-*α* level and a significant decrease (*p* > 0.05; -67.19%) in serum IL-4 level. As a result of treatment of APAP-administered rats with the peel hydroethanolic extract, naringin, and naringenin, the elevated serum TNF-*α* level was significantly (*p* > 0.05) improved recording percentage changes of -35.26, -34.15, and -36.46%, respectively, as compared with APAP-administered control. Conversely, the serum IL-4 level was significantly increased (*p* > 0.05) as a result of treatments with the peel extract, naringin, and naringenin recording percentage increases of 93.04, 133.97, and 114.59, respectively, as compared with APAP-administered control (Table 4).

### 3.6. Histopathological Effects

**Figure 3:**
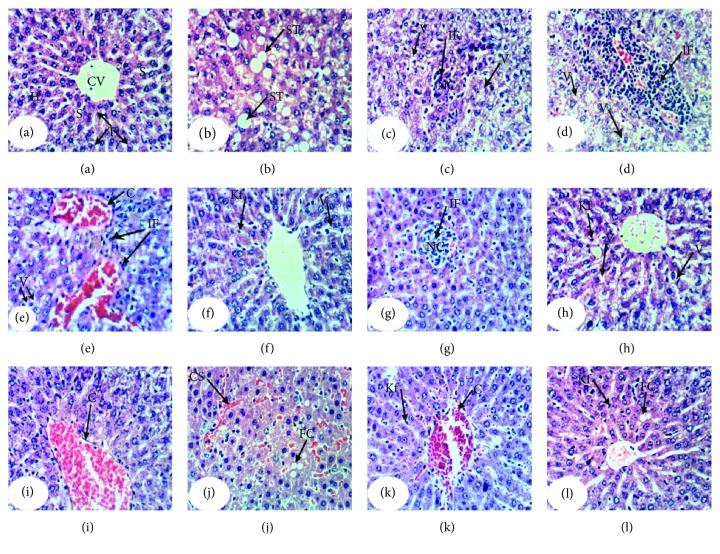
Photomicrographs of H&E-stained liver sections of normal, APAP-administered rats, and APAP-administered rats treated with the navel orange peel hydroethanolic extract, naringin, and naringenin. (a) Photomicrograph of the liver of normal rat showing a normal structure of liver; normal hepatocytes (H) integrally arranged in the hepatic trabeculae, central vein (CV), and sinusoids (S). (b-e) Photomicrographs of liver sections of APAP-administered rats showing steatosis of hepatocytes (ST) in (b), cytoplasmic vacuolization of hepatocytes (V) and focal hepatic necrosis (NC) associated with mononuclear leucocytic and fibroblastic inflammatory cell infiltration (IF) in (c), portal mononuclear leucocytic inflammatory cell infiltration (IF) in (d), and vascular congestion in (e). (f & g) Photomicrographs of liver sections of APAP-administered rats treated with the navel orange peel hydroethanolic extract showing slight cytoplasmic vacuolization of hepatocytes (V) and Kupffer cell activation (Kf) and mild focal hepatic necrosis (NC) associated with mononuclear leucocytic inflammatory cell infiltration (IF). (h & i) Photomicrographs of liver sections of APAP-administered rats treated with naringin showing severe to mild cytoplasmic vacuolization of hepatocytes (V) and Kupffer cell activation (Kf) in (h) and (i) as well as congestion of the central vein (C) in (h). (j-l) Photomicrographs of liver sections of APAP-administered rats treated with naringenin showing a slight fatty change of hepatocytes (FC) and mild congestion of sinusoids (CS) in (j), Kupffer cell activation (Kf) and moderate congestion of central vein (C) in (k), and slight fatty change of hepatocytes (FC) and Kupffer cell activation (Kf) in (l) (×400).

Histopathological examination of the liver sections of normal control rats showed normal histological architecture (Figure 3; photomicrograph a). On the other hand, APAP administration produced liver histological changes and several lesions including steatosis of hepatocytes, cytoplasmic vacuolization of the hepatocytes, focal hepatic necrosis linked with mononuclear leucocytic and fibroblastic inflammatory cells' infiltration, and portal infiltration with mononuclear leucocytic inflammatory cells (Figure 3; photomicrographs b–e). The treatment of APAP-administered rats with the navel orange peel hydroethanolic extract produced an amelioration of the hepatic tissue with slight cytoplasmic vacuolization of hepatocyte, Kupffer cell activation, and focal hepatic necrosis associated with mononuclear leucocytic inflammatory cell infiltration (Figure 3; photomicrographs f & g). Also, the treatment with naringin resulted in slight cytoplasmic vacuolization of hepatocytes, Kupffer cell activation, and congestion of the central vein (Figure 3; photomicrographs h & i). Administration of naringenin to APAP-administered rats produced a mild fatty change of hepatocytes, congestion of the central vein and sinusoids, and Kupffer cell activation (Figure 3; photomicrographs j, k, & l). Accordingly, the liver histological integrity and architecture of APAP-administered rats were markedly improved as a result of treatments with the navel orange peel hydroethanolic extract, naringin, and naringenin.

Histopathological change scores of study groups are represented in Table 5. The liver section of normal control rats exhibited no histological lesions recording zero score. The liver APAP-administered rats exhibited various grades of histopathological change scores ranging from grade III to grade 0. The treatments of APAP-administered rats with the navel orange peel hydroethanolic extract, naringin, and naringenin resulted in marked improvements in liver histological lesions including steatosis or fatty changes, inflammatory cell infiltration, necrosis, hydropic degeneration, vacuolar degeneration, vascular congestion, and Kupffer cell proliferation (activation) exhibiting lower grades of histopathological change scores in most of treated animals as compared with APAP-administered control.

### 3.7. Effect on Immunohistochemically Stained Liver p53, Bax, Caspase-3, and Bcl-2

**Figure 4:**
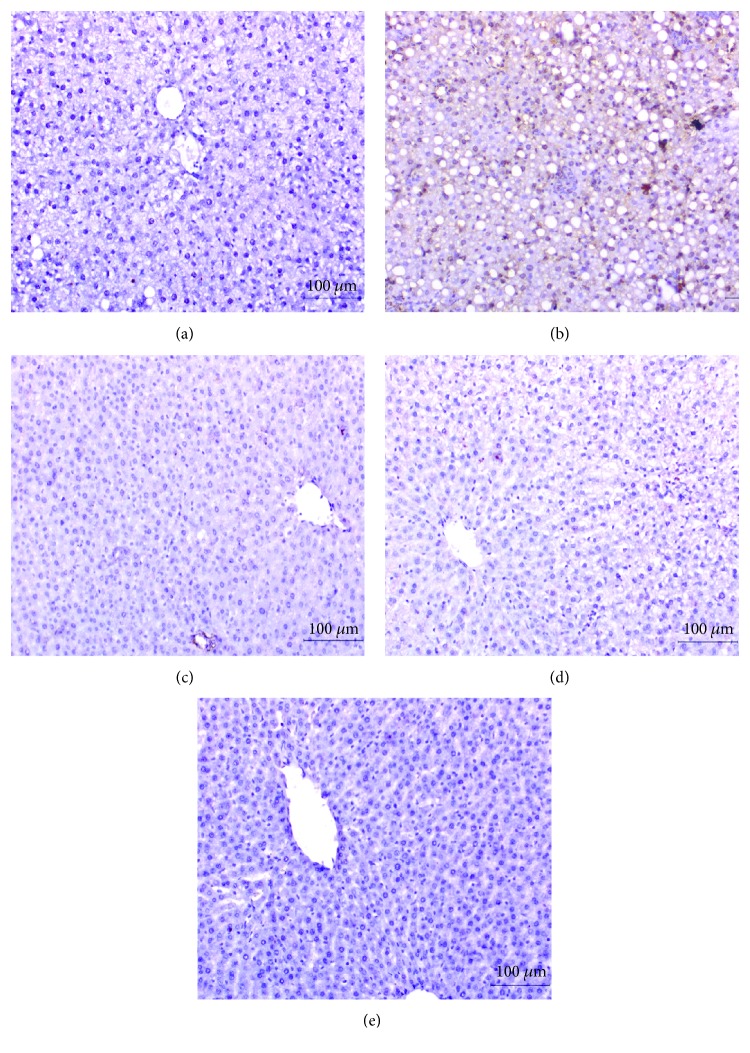
Immunohistochemically stained liver sections for p53 expression detection. (a) Negative (-) or very weak immunohistochemical reaction in normal control. (b) Strong positive immunohistochemical reaction marked by a dense brown colour in the cytoplasm and nucleus of hepatocytes in APAP-administered rats. (c-e) Substantial decrease in the expression of p53 in APAP-administered rats treated with the navel orange peel extract (c), naringin (d), and naringenin (e) (×200).

**Figure 5:**
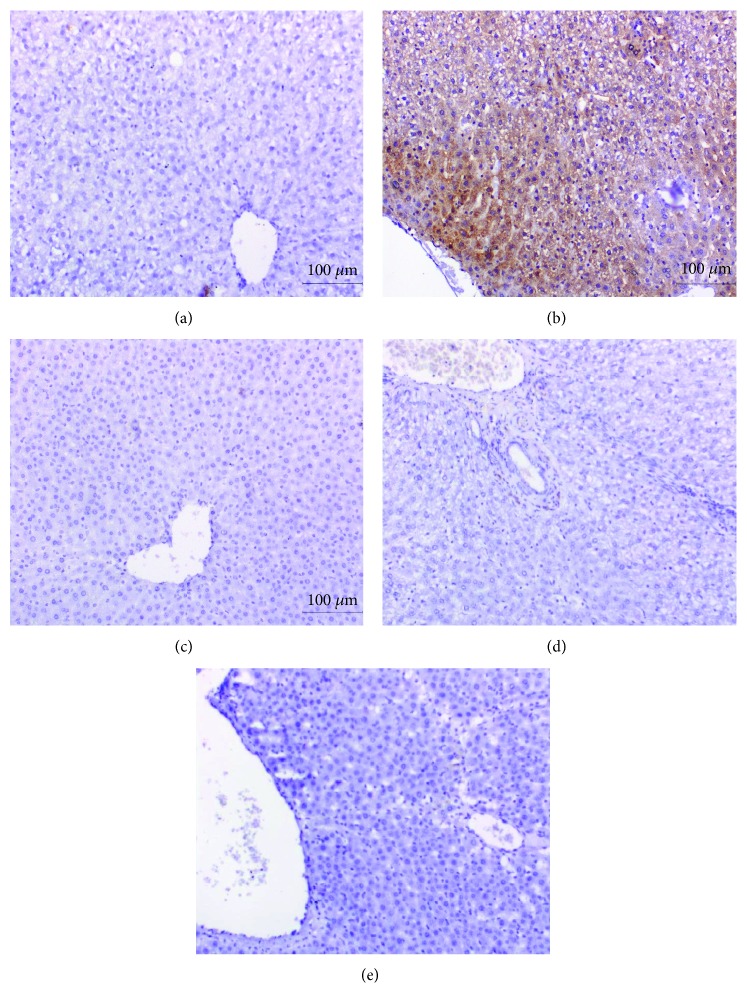
Immunohistochemically stained liver sections for Bax expression detection. (a) Negative or very weak immunohistochemical reaction in normal control. (b) Evoked a strong positive immunohistochemical reaction marked by a dense brown colour in the cytoplasm of hepatocytes in APAP-administered rats. (c-e) Profound suppression of Bax expression in APAP-administered rats treated with the navel orange peel extract (c), naringin (d), and naringenin (e) (×200).

**Figure 6:**
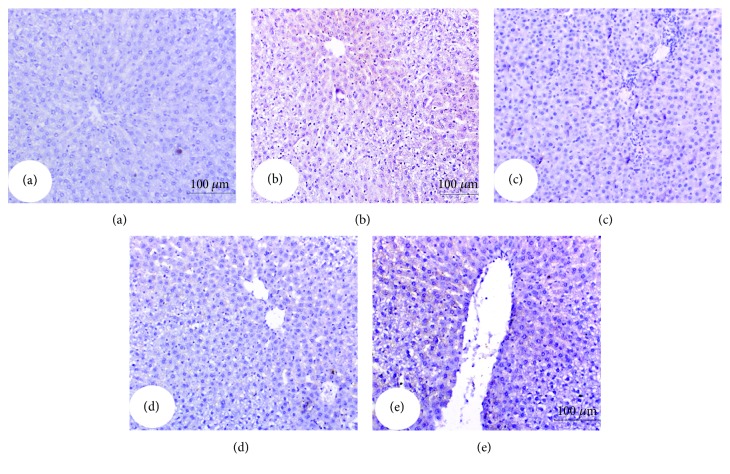
Immunohistochemically stained liver sections for caspase-3 expression detection. (a) Negative or very weak immunohistochemical reaction in normal control. (b) Strong immunohistochemical reaction marked by a dark brown colour in hepatocytes in the liver of APAP-administered rats. (c-e) Low expression of caspase-3 in APAP-administered rats treated with the navel orange peel extract (c) and naringin (d) and moderate immunohistochemical reaction in APAP-administered rats treated with naringenin (e) (×200).

**Figure 7:**
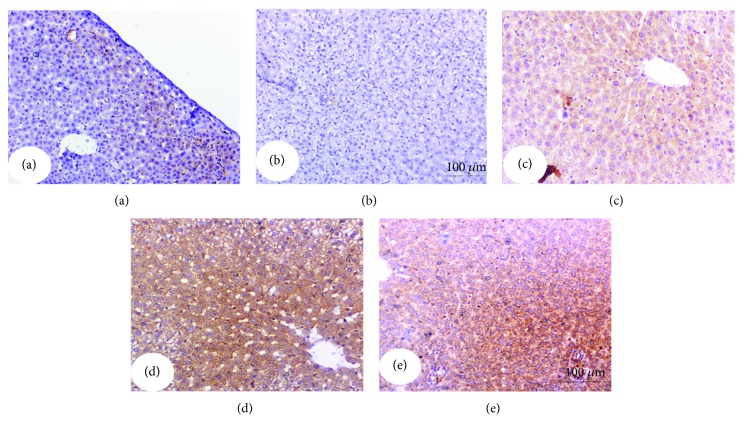
Immunohistochemically stained liver sections for Bcl-2 expression detection. (a) Moderate immunohistochemical reaction in normal control. (b) Very low expression in hepatocytes in the liver of APAP-administered rats. (c-e) Moderate, very strong, and strong immunohistochemical reactions for Bcl-2 in the cytoplasm of APAP-administered rats treated with the navel orange peel extract (c), naringin (d), and naringenin (e), respectively (×200).

The expressed p53, Bax, and activated caspase-3 as well as Bcl-2 in the liver were immunohistochemically detected (Figures 4–7 and Table 6). Immunohistochemical staining of the liver section of normal control rats exhibited a very weak immunohistochemical reaction for p53, Bax, and caspase-3 reflecting their very low expression. The liver of APAP-administered rats exhibited a strong positive staining for these proapoptotic markers as indicated by a dense brownish colour reflecting their very high expression. The liver of APAP-administered rats treated with the navel orange peel hydroethanolic extract, naringin, and naringenin exhibited a substantial decrease of p53, Bax, and caspase-3 (Figures 4–6 and Table 6). While the treatment with naringin was the most potent in decreasing p53 and caspase-3, naringenin was the most effective in decreasing Bax. In contrast, Bcl-2 exhibited a different behavioural pattern to p53, Bax, and caspase-3 since it exhibited a profound depletion of antiapoptotic protein Bcl-2 in APAP-administered rats (Figure 7). On the other hand, the liver of APAP-administered rats treated with the navel orange peel extract, naringin, and naringenin exhibited a very strong reaction reflecting a very high expression of Bcl-2. According to the intensity of the brown colour in Figure 7 and results of intensities in Table 6, the treatments were arranged in the following order: naringin>naringenin>navel orange peel extract.

## 4. Discussion

APAP, when used at high doses, could cause acute liver injury most probably *via* formation of NAPQI, a toxic oxidant, by cytochrome P450 [53]. NAPQI is usually inactivated by hepatic GSH, but when produced excessively, it covalently binds to centrilobular hepatic proteins and depletes GSH content, contributing to hepatic toxicity [54].

To assess the effects APAP on liver function and integrity, in the present study, the activities of serum ALT, AST, ALP, and GGT and levels of bilirubin and albumin were determined and the histological changes in the liver were examined. Oral administration of APAP, in the present study, at a dose of 0.5 g/kg/b.w. every other day for 4 weeks, showed a sharp increase in serum ALT, AST, ALP, LDH, and GGT activities. These results are in parallel with those of Hanafy et al. [19], Tan et al. [55], and Tung et al. [20]. The massive presence of NAPQI causes mitochondrial GSH depletion, the formation of protein adducts, with severe damages to mitochondrial functions, and the arrest of ATP production [56]. All these modifications result in a homeostasis alteration and an increase in the permeability of the cell membrane with a consequent cellular swelling, vacuolization, karyolysis, and the loss of cellular elements, thereby leading to hepatocyte damage and necrosis [11]. So, the increase in serum activities of the hepatic enzymes in APAP-administered rats, in this study, may be attributed to the structural damage of hepatocytes resulting in the leakage of cytosolic enzymes (AST, ALT, and LDH) and release of membrane-bound enzymes (ALP and GGT) from the liver into the systemic circulation [18, 57]. ALP and GGT are membrane-bound enzymes, and their increase may be due to the stimulated rate of synthesis and/or regurgitation into the blood as a result of bile ductule blockage [58]. The increase in serum total bilirubin level confirmed the suggestion that ALP and GGT levels are increased following hepatic necrosis due to secondary biliary obstructions [59]. The present histological results support this attribution since they indicated hepatic portal mononuclear leucocytic inflammatory cells' infiltration which may lead to bile ductule blockage and biliary obstructions in association with hepatocytes' cytoplasmic vacuolization and necrosis in APAP-administered rats. This blockage of bile ductules leads to regurgitation of conjugated and unconjugated bilirubin into blood circulation and in turn results in elevated total bilirubin levels.

In contrast to the serum total bilirubin level, the serum albumin level was remarkably decreased in APAP-administered rats and this result is in concurrence with Okokon et al. [56]. This reduction in albumin level in APAP-administered rats could be due to a decline in the number of cells responsible for albumin synthesis in the liver through necrosis and degeneration [60]. A decreased level of albumin, as recorded in APAP-administered rats, revealed the severity of hepatopathy [56].

Peels of fruits of *Citrus* species are known to have antioxidant, anti-inflammatory, anticancer, antilipidemic, and antibacterial activities [61]. The *citrus* flavonoids include a class of glycosides such as hesperidin and naringin and another class of O-methylated aglycones of flavones like nobiletin and tangeretin, which are relatively two common polymethoxylated flavones (PMFs) [62]. The *Citrus* fruit peels are reported to possess the highest amounts of PMFs compared to other edible parts of the fruit [63].

The navel orange (*C. sinensis*) peel hydroethanolic extract contains rich phytochemicals as indicated in the present study by GC-MS analysis. Many of these phytochemicals have potent biological activities. 4H-pyran-4-one, detected at a retention time of 16.365 minutes, is a cyclic nucleus in the chemical structures of quercetin, naringenin, naringin, and hesperetin which have multiple biological properties such as hepatoprotective, antioxidant, antibacterial, antiviral, anticancer, and anti-inflammatory effects [64]. 4H-Pyran-4-one, 2,3-dihydro-3,5-dihydroxy-6-methyl- (1.452% of the extract) was reported to have a strong free radical scavenging activity [65, 66]. Thymine, detected at a retention time of 15.179 minutes, is a nitrogenous base component in the nucleic acid of DNA [67]. 5-Hydroxymethylfurfural and 4-hexen-3-one, 4,5-dimethyl-, detected at a retention time of 17.470 minutes, constitute 6.392% of the navel orange peel extract. 5-Hydroxymethylfurfural was demonstrated by Ding et al. [68] and Zhao et al. [69] to have cancer preventive, antioxidant, and hepatic and renal protective effects. Dodecane, detected at retention times 24.018 and 33.851 with abundance of 7.392 and 1%, respectively, was elucidated to have potent antioxidant activity [70]. 2-Methoxy-4-vinylphenol- and 3-methoxyacetophenone were detected at retention time 19.793 with abundance of 2.715% in the peel hydroethanolic extract. 2-Methoxy-4-vinylphenol- was stated to have antioxidant, antimicrobial, and anti-inflammatory properties [71] while 3-methoxyacetophenone has antimycobacterial activity [72]. 9,12-Octadecadienoic acid (Z,Z)-, detected at a retention time of 34.949 minutes with abundance of 1.151% in the extract, was found to have potential cancer preventive, hepatoprotective, hypocholesterolemic, anti-inflammatory, and antiarthritic activities [73]. Oleic acid and 9-octadecenoic acid, (E)- (2.755% of the extract), detected at a retention time of 34.998 minutes, were reported to have antitumor and anti-inflammatory activities [73, 74]. Lupanine (10.225% of the extract), detected at retention times of 36.064 and 36.135 minutes, is a quinolizidine alkaloid that was reported to have antimicrobial effects [75]. Stigmasterol (2.028% of the extract; detected at a retention time of 36.885 minutes) and its derivatives have several pharmacological activities including antidiabetic, anti-inflammatory, antioxidant, antimutagenic, and antimicrobial effects [76]. 9-Octadecenamide, (Z)- (1.568% of the extract; detected at a retention time of 37.244 minutes), was revealed to have anti-inflammatory and antibacterial activities [77].

The HPLC-MS analysis of the navel orange peel hydroethanolic extract confirmed the presence of phenolic acids such as gallic acid and flavonoids such as diosmin, naringin, rutin, hesperidin, quercetin, naringenin, and hesperetin. Hesperidin, quercetin, and naringenin were the most abundant constituents in the peel extract.

Oral treatment of APAP with the navel orange peel hydroethanolic extract induced a hepatoprotective effect on liver function marked by ameliorating the activities of hepatic enzymes and levels of albumin and bilirubin in serum. These data are in parallel with Chen et al. [78] who indicated that the administration of the sweet orange peel extract at 10 and 100 mg/kg b.w. reduces the hepatotoxicity induced by CCl_4_. The hepatoprotective effect of *Citrus*by-product extracts may be due to the presence of phytoconstituents like phenolic compounds, especially the characteristic flavanone glycosides that mainly include naringin, hesperidin, neohesperidin, and narirutin [79]. The improvement of the liver function of APAP-administered rats as a result of treatments with naringin and naringenin, in the current study, supports this attribution. This improvement was manifested by a significant decrease in serum ALT, AST, ALP, LDH, and GGT activities and total bilirubin level together with the significant increase in serum albumin level after treatment of APAP-administered rats with naringin and naringenin.

In the present study, the ingestion of APAP to rats induced a marked disturbance in the oxidant/antioxidant status characterized by a significant increase in liver LPO and a significant decrease in hepatic GSH content and GPx, GST, and SOD activities. These findings are in accordance with those of Amin et al. [80], Tan et al. [55], and Tung et al. [20]. This increase in oxidative stress may be mediated, at least in part, via accumulation of the toxic metabolite NAPQI, a metabolite of APAP that has high affinity for GSH leading to its depletion [80].

According to a previous report, the hepatoprotective effect of the compounds is related, at least in part, to their antioxidant activity [81]. Antioxidants have been shown to prevent oxidative stress-related liver pathologies directly, by scavenging of ROS, and indirectly, as part of the antioxidant defense system [82]. The flavonoids, which play a role as free radical scavengers or antioxidants in biological systems, are natural phenolic compounds present in fruit and vegetable species [83]. Flavonoids have many biological effects such as antioxidant, antibacterial, anticancer, antimutagenic, and anti-inflammatory properties [84].

In the current study, the treatment of rats with the navel orange peel hydroethanolic extract for 4 weeks significantly decreased the elevated liver LPO and markedly increased the liver GSH content and GST, GPx, and SOD activities. These ameliorative effects might be due to the potential antioxidant activities of the constituting flavonoids and other components of the hydroethanolic extracts. These results are in concurrence with those obtained by Mostafa et al. [27].

The wide range of biological functions of flavonoids in orange peel has been extensively and recently studied. Such flavonoids increased the antioxidant capacity against lipid peroxidation and reduced the elderly oxidative stress [85]. The antioxidant activity found in the peels and seeds of *Citrus sinensis* could be attributed to the presence of phenolic compounds as evidenced by Molan et al. [86]. In the same regard, Chen et al. [78] observed that the pretreatment with the sweet orange peel extract significantly attenuated the elevation of LPO and prevented the oxidative damage induced by CCl_4_ in rats.

In view of the current study, APAP-induced oxidative stress was significantly suppressed by naringin and naringenin treatment *via* reduction of lipid LPO and enhancement of the activities of antioxidant enzymes, which in turn modulate the membrane integrity against APAP-induced cellular injury. These data are in accordance with Adil et al. [30].

Naringenin's protective effect could be explained by its important role in preventing hydroxyl radical formation and protecting the integrity and functions of tissues, like other antioxidant elements [87, 88]. The hepatopreventive effect of the navel orange peel hydroethanolic extract, naringin, and naringenin may be explained by their ability to increase the antioxidant enzymes and to suppress LPO.

In the present study, the navel orange peel extract was more effective in improving the liver LPO, GSH content, and GPx activity than were naringin and naringenin. This may be due to the fact that the navel orange peel hydroethanolic extract contains many antioxidant polyphenols and phytochemicals, as confirmed by GC-MS and HPLC analyses in the present study, which may synergize to produce this most potent effect.

**Figure 8:**
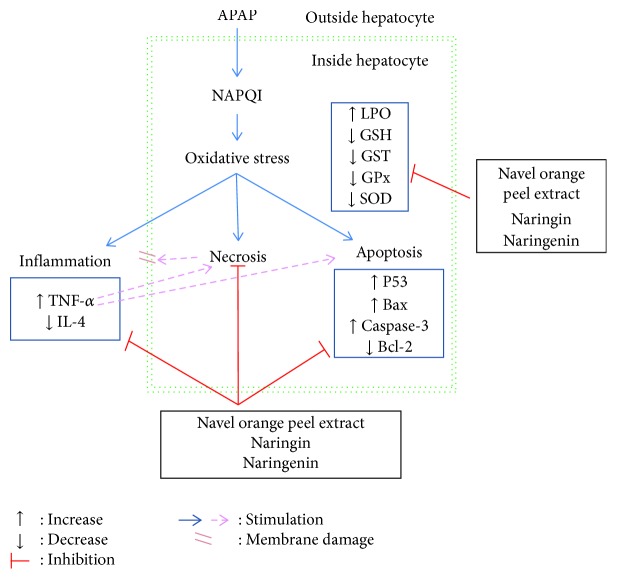
Schematic diagram of APAP-induced hepatotoxicity and its prevention by the navel orange peel hydroethanolic extract, naringin, and naringenin.

Concerning the effects on inflammatory status, the oral administration of APAP significantly increased the serum proinflammatory cytokine (TNF-*α*) and decreased anti-inflammatory interleukin (IL-4). TNF-*α* has been linked to increased oxidative stress (Figure 8) and is known to recruit and activate other inflammatory cells [89]. The treatments of APAP-administered rats with the navel orange peel hydroethanolic extract, naringin, and naringenin, in the current study, ameliorated the elevated TNF-*α* and the decreased IL-4 levels. In parallel to these results, naringin was reported to reduce inflammation by inhibiting NF-*κ*B in lipopolysaccharide-induced acute lung injury model in mice [90]. Moreover, Chtourou et al. [91] stated that naringenin is able to decrease proinflammatory cytokines such as TNF-*α*, IL-6, and IL-1*β* in rats *via* inhibition of NF-*κ*B, a signal transduction pathway that promotes the transcription of gene coding for proinflammatory proteins. Similarly, Dou et al. [92] have demonstrated that naringenin also has anti-inflammatory efficacies in the gut.

In the present study, the results of immunohistochemical investigations showed overexpression of proapoptotic proteins (p53, Bax, and activated caspase-3) and a negative or very weak immunohistochemical reaction to antiapoptotic protein (Bcl-2) in the liver of APAP-administered rats. On the other hand, the treatment of APAP-administered rats with the navel orange peel hydroethanolic extract, naringin, and naringenin suppressed the expression of p53, Bax, and caspase-3 and caused overexpression of Bcl-2 in the liver tissue. Naringin was the most effective in decreasing the APAP-induced expression of liver p53 and caspase-3 and in increasing the expression of Bcl-2 while naringenin was the most potent in decreasing Bax overexpression. In our opinion, the inhibitory effects of the navel orange peel hydroethanolic extract, naringin, and naringenin on APAP-induced apoptosis may be due to their antioxidant properties and their ability to scavenge free radicals (Figure 8). This attribution was supported by previous publications which indicated that the treatment of APAP-administered animals with ROS scavengers such as fucoidan and coenzyme Q10 successfully prevented the APAP-induced overexpression of p53, caspase-3, and Bax as well as APAP-induced downregulation of Bcl-2 [16, 93]. However, our study is in disagreement with Sun et al. [94] who elucidated that overexpression of p53 prevents APAP-induced liver injury by regulating the metabolizing enzymes and transporters related to APAP detoxification and elimination.

The p53 tumor suppressor protein is a critical regulator of the cell cycle and apoptosis, and it is considered as a proapoptotic mediator [17]. In most unstressed cells, p53 is expressed at low levels in both the nucleus and cytoplasm [95]. But it is activated due to DNA damage and other stresses such as excessive ROS production and inflammation as in APAP hepatotoxicity [96, 97]. Moreover, previous studies reported the involvement of caspase activation in p53-mediated cell death [98, 99]. In this way, p53 overexpression may stimulate caspase-3 and cleavage of caspase substrates possibly through caspase-8 (extrinsic pathway or death receptor pathway that is initiated by death ligands such as TNF-*α*) and caspase-9 (mitochondrial or intrinsic pathway) activation pathways [97, 100]. In the intrinsic pathway, p53 induces the release of mitochondrial cytochrome *c* by a pathway involving cytosolic Bax translocated to mitochondria (outer membrane) leading to activation of caspase-9 and caspase-3 and thereby apoptosis [97, 98, 100–103]. Death receptors, such as tumor necrosis factor receptor 1, by the caspase-8 cleavage of Bid and activation of Bax, can also induce mitochondrial cytochrome *c* release that activates caspase-3 [100]. It was suggested that APAP exposure in rats increased the Bax level and decreased Bcl-2, leading to permeabilization of the mitochondrial outer membrane, cytochrome c release, activation of caspase-3, & endonuclease G and DNA fragmentation [104, 105]. Thus, caspase-3, cleaved from caspase-8, caspase-9, and caspase-10, serves as a convergence point for different signaling pathways; thereby, it is well suited as a readout in an apoptosis assay and its increased expression reflects an increase in apoptosis [106]. Based on our results and the previous literature, the activation of caspase-3, the executioner of apoptosis, in APAP hepatotoxicity can be triggered either by the intrinsic pathway through excess levels of ROS or by extrinsic pathway death receptor ligands as TNF-*α* (Figure 8). It is relevant here to mention that TNF-*α*-induced cell death is predominantly apoptotic but may also occur by necrosis (Figure 8) [107]. The treatment of APAP-administered rats with the navel orange peel hydroethanolic extract, naringin, and naringenin potentially suppressed the oxidative stress and significantly decreased the elevated TNF-*α* level; thereby, they decreased apoptosis through affecting both extrinsic and extrinsic apoptotic pathways (Figure 8).

## 5. Conclusion

The navel orange peel hydroethanolic extract, naringin, and naringenin induced potential hepatopreventive effects which were evidenced by amelioration of the liver function and histological integrity, liver antioxidant activities, and serum proinflammatory and anti-inflammatory cytokine levels in addition to alleviation of apoptotic markers. Thus, the ameliorative effects of the navel orange peel hydroethanolic extract, naringin, and naringenin may be mediated *via* enhancement of the antioxidant defense system and suppression of inflammation and apoptosis.

## Figures and Tables

**Table 1:** Chemical composition of the navel orange hydroethanolic extract as detected by GC-MS analysis.

Number	Retention time	Compound (from the Central Library search report)	Area % (higher than 1%)
1	15.179	(i) Delta 2-tetrazaboroline, 5-ethyl-, 1,4-dimethyl- (ii) 4,5-Diamino-2-hydroxypyrimidine (iii) Thymine	1.324%
2	16.395	(i) 4H-Pyran-4-one, 2,3-dihydro-3,5-di hydroxy-6-methyl-	1.452%
3	17.470	(i) 2-Butanone, 4-hydroxy-3-methyl- (ii) Dimethylamine, N-(neopentyloxy)-	1.382%
4	17.607	(i) No matches in library	2.379%
5	18.295	(i) 5-Hydroxymethylfurfural (ii) 4-Hexen-3-one, 4,5-dimethyl-	6.392%
6	19.793	(i) 2-Methoxy-4-vinylphenol (ii) 3-Methoxyacetophenone	2.715%
7	23.252	(i) No matches in library	3.271%
8	23.944	(i) No matches in library	10.524%
9	24.018	(i) Dodecane	7.392%
10	25.034	(i) No matches in library	1.364%
11	27.179	(i) No matches in library	10.687%
12	32.867	(i) 3-Deoxy-d-mannoic lactone (ii) Beta-D-Glucopyranose, (iii) 4-O-beta-D-galactopyranosyl- (iv) Sucrose	1.377%
13	33.851	(i) Dodecane	1.00%
14	34.949	(i) 9,12-Octadecadienoic acid (Z,Z)- (ii) cis-7-Dodecen-1-yl acetate	1.151%
15	34.998	(i) Oleic acid (ii) 9-Octadecenoic acid, (E)-	2.755%
16	36.064	(i) Lupanine	4.380%
17	36.135	(i) Lupanine	5.845%
18	36.885	(i) Stigmasterol (ii) 1-Methyl-4-(1-methylethenyl)-3-[1-methyl-1-(4-methyl-hex-5-enyl)-5,9-dimethyldec-4-enyl] cyclohexene. (iii) 26,27-Dinorergosta-5,23-dien-3-ol, (3beta)-	2.028%
19	37.244	(i) 9-Octadecenamide, (Z)-	1.568%

**Table 2:** Effects of the navel orange peel hydroethanolic extract, naringin, and naringenin on serum ALT, AST, ALP LDH, and GGT activities and albumin and total bilirubin levels in APAP-administered Wistar rats.

Parameter	ALT (U/l)	AST (U/l)	ALP (U/l)	LDH (U/l)	GGT (U/l)	Albumin (g/dl)	Total bilirubin (mg/dl)
Treatments							
Normal control	30.33 ± 0.91	66.66 ± 1.542	448.00 ± 17.03	1776.81 ± 42.56	3.50 ± 0.43	3.23 ± 0.08	0.70 ± 0.05
APAP	86.16 ± 2.99^a^	141.50 ± 2.591^a^	757.50 ± 19.53^a^	3522.50 ± 98.20^a^	9.88 ± 0.32^a^	2.71 ± 0.07^a^	1.31 ± 0.13^a^
APAP + navel orange peel extract	33.33 ± 2.27^b^	78.83 ± 3.218^b^	492.16 ± 23.73^b^	1825.01 ± 43.93^b^	3.50 ± 0.43^b^	3.20 ± 0.07^b^	0.72 ± 0.07^b^
APAP + naringin	34.33 ± 2.09^b^	73.83 ± 4.053^b^	497.83 ± 27.83^b^	1869.80 ± 32.57^b^	3.66 ± 0.33^b^	3.20 ± 0.09^b^	0.73 ± 0.08^b^
APAP + naringenin	35.50 ± 1.66^b^	74.16 ± 3.807^b^	483.50 ± 32.51^b^	1852.02 ± 29.92^b^	2.66 ± 0.33^b^	3.23 ± 0.09^b^	0.71 ± 0.09^b^

Data are expressed as mean ± SE of *n* = 6. ^a^Significantly different from the normal value at *p* < 0.05. ^b^Significantly different from the APAP value at *p* < 0.05.

**Table 3:** Effects of the navel orange peel hydroethanolic extract, naringin, and naringenin on liver LPO and GSH level of APAP-administered Wistar rats.

Parameter	LPO (nmol/100 mg tissue)	GSH (nmol/10 mg tissue)	GST (U/100 mg tissue)	GPx (mU/100 mg tissue)	SOD (mU/100 g tissue)
Treatments					
Normal control	53.66 ± 1.11	31.68 ± 0.67	44.73 ± 1.11	185.26 ± 1.19	99.97 ± 1.32
APAP	112.45 ± 1.89^a^	20.81 ± 0.47^a^	34.25 ± 0.80^a^	169.53 ± 2.37^a^	68.34 ± 1.63^a^
APAP + navel orange peel extract	72.54 ± 3.09^ab^	27.30 ± 1.01^b^	36.18 ± 1.71^a^	180.66 ± 1.88^b^	89.15 ± 1.77^ab^
APAP + naringin	82.85 ± 5.74^ab^	26.26 ± 2.05^ab^	40.80 ± 1.86^b^	179.23 ± 2.02^b^	72.46 ± 1.22^a^
APAP + naringenin	88.05 ± 7.11^ab^	26.03 ± 1.06^ab^	39.22 ± 1.18	179.03 ± 1.73^b^	89.07 ± 2.44^ab^

Data are expressed as mean ± SE of *n* = 6. ^a^Significantly different from the normal value at *p* < 0.05. ^b^Significantly different from the APAP value at *p* < 0.05.

**Table 4:** Effects of the navel orange peel ethanolic extract, naringin, and naringenin on serum TNF-*α* and IL-4 levels of APAP-administered Wistar rats.

Parameter	TNF-*α* (pg/ml)	IL-4 (pg/ml)
Treatments		
Normal control	33.26 ± 1.56	122.63 ± 1.72
APAP	131.46 ± 1.91^a^	40.23 ± 1.53^a^
APAP + navel orange peel extract	85.10 ± 4.75^ab^	77.66 ± 5.28^ab^
APAP + naringin	86.56 ± 6.67^ab^	94.13 ± 6.18^ab^
APAP + naringenin	83.53 ± 1.58^ab^	86.33 ± 6.85^ab^

Data are expressed as mean ± SE of *n* = 6. ^a^Significantly different from the normal value at *p* < 0.05. ^b^Significantly different from the APAP value at *p* < 0.05.

**Table 5:** Histopathological scores of liver lesions in normal control, APAP, APAP + peel extract, APAP + naringin, and APAP + naringenin groups.

Histopathological changes	Score	Normal control	APAP	APAP + peel extract	APAP + naringin	APAP + naringenin
Steatosis and fatty changes	0	6 (100%)	1 (16.7%)	5 (83.3%)	5 (83.3%)	5 (83.3%)
	I	—	1 (16.7%)	—	1 (16.7%)	1 (16.7%)
	II	—	2 (33.3%)	—	—	—
	III	—	2 (33.3%)	1 (16.7%)	—	—

Inflammation	0	6 (100%)	1 (16.7%)	4 (66.7%)	5 (83.3%)	5 (83.3%)
	I	—	1 (16.7%)	1 (16.7%)	—	—
	II	—	2 (33.3%)	1 (16.7%)	1 (16.7%)	1 (16.7%)
	III	—	2 (33.3%)	—	—	—

Necrosis	0	6 (100%)	1 (16.7%)	4 (66.7%)	4 (66.7)	5 (83.3%)
	I	—	1 (16.7%)	2 (33.3%)	1 (16.7%)	1 (16.7%)
	II	—	3 (50%)	—	1 (16.7%)	—
	III	—	1 (16.7)	—	—	—

Hydropic degeneration	0	6 (100%)	3 (50.0%)	6 (100%)	6 (100%)	6 (100%)
	I	—	—	—	—	—
	II	—	1 (16.7%)	—	—	—
	III	—	2 (33.3%)	—	—	—

Vacuolar degeneration	0	6 (100%)	—	4 (66.7%)	1 (16.7%)	6 (100%)
	I	—	1 (16.7%)	1 (16.7%)	2 (33.3%)	—
	II	—	1 (16.7%)	1 (16.7%)	1 (16.7%)	—
	III	—	4 (66.7%)	—	1 (16.7%)	—

Vascular congestion	0	6 (100%)	2 (33.3%)	5 (83.3%)	4 (66.7%)	4 (66.7%)
	I	—	2 (33.3%)	1 (16.7%)	1 (16.7%)	1 (16.7%)
	II	—	1 (16.7%)	—	—	1 (16.7%)
	III	—	1 (16.7%)	—	1 (16.7%)	—

Kupffer cell proliferation	0	6 (100%)	2 (33.3%)	4 (66.7%)	4 (66.7%)	3 (15.0%)
	I	—	1 (16.7%)	1 (16.7%)	2 (33.3%)	1 (16.7%)
	II	—	1 (16.7%)	1 (16.7%)	—	1 (16.7%)
	III	—	2 (33.3%)	—	—	1 (16.7%)

0: absence of lesion; I: mild; II: moderate; and III: severe. The number of animals in each group is 6. The % in parentheses is the percent of animals in each grade.

**Table 6:** Immunohistochemical staining intensity (pixels) for p53, Bax, caspase-3, and Bcl-2 in liver normal control, APAP, APAP + peel extract, APAP + naringin, and APAP + naringenin groups.

Parameter	p53 × 10^3^	Bax × 10^3^	Caspase − 3 × 10^3^	*Bcl* − 2 × 10^3^
Group				
Normal control	2.125 ± 0.686	0.379 ± 0.119	0631 ± 0.046	10.18 ± 0.585
APAP	69.277 ± 3.075^a^	91.585 ± 14.270^a^	15.426 ± 2.467^a^	0.580 ± 0.087^a^
APAP + peel extract	1.007 ± 0257^b^	0.497 ± 0.096^b^	0.697 ± 0.157^b^	125.57 ± 11.012^b^
APAP + naringin	0.828 ± 0123^b^	0.387 ± 0.116^b^	0.482 ± 0.054^b^	197.505 ± 33.008^b^
APAP + naringenin	1.653 ± 0424^b^	0.207 ± 0.126^b^	1.740 ± 0.366^b^	190.504 ± 9.905^b^

Data are expressed as mean ± SE. ^a^Significantly different from normal value at *p* < 0.05. ^b^Significantly different from the APAP value at *p* < 0.05.

## Data Availability

All data used in this article are publicly available and accessible online.

## Abbreviations

ALP: Alkaline phosphatase
ALT: Alanine transaminase
APAP: N-Acetyl-p-aminophenol
AST: Aspartate transaminase
Bcl-2: B-cell lymphoma-2
CMC: Carboxymethyl cellulose
DAD: Diode array detector
GC-MS: Gas chromatography-mass spectrometry
GGT: Gamma-glutamyl transferase
GPx: Glutathione peroxidase
GSH: Glutathione
GST: Glutathione-S-transferase
HPLC-MS: High-performance liquid chromatography-mass spectrometry
IL-4: Interleukin-4
iNOS: Inducible nitric oxide synthase
LDH: Lactate dehydrogenase
LPO: Lipid peroxidation
NAPQI: N-Acetyl-p-benzoquinone imine
NF-*κ*B: Nuclear factor-kappa B
p53: Protein 53
r.p.m.: Rounds per minute
ROS: Reactive oxygen species
RT-PCR: Reverse transcriptase-polymerase chain reaction
SOD: Superoxide dismutase
TNF-*α*: Tumor necrosis factor-*α*.
